# Autografted Electrical Burn Complicated by Cutaneous Chromoblastomycosis

**Published:** 2015-11-03

**Authors:** Derek Erstad, Naomi Sell, Ryan Cauley, Zehra Sahin, Jeremy Goverman

**Affiliations:** ^a^Department of Surgery, Massachusetts General Hospital, Boston; ^b^Division of Burn Surgery, Massachusetts General Hospital, Boston; ^c^Department of Pathology, Massachusetts General Hospital, Boston

**Keywords:** chromoblastomycosis, verrucous dermatitis, subcutaneous mycoses, dematiaceous fungi, cutaneous ulcer

## DESCRIPTION

An 84-year-old man from the Dominican Republic presented with an 8-year history of a chronic forearm wound from an electrical burn that was complicated by chromoblastomycosis infection and was initially treated with excision and autografting in 2013. He now presented 2 years later with verrucous masses over the graft site, determined to be chromoblastomycosis recurrence.

## QUESTIONS

1. **What are the clinical features of chromoblastomycosis and what is the associated differential diagnosis?**
2. **What is the treatment of chromoblastomycosis?**
3. **How is chromoblastomycosis diagnosed?**
4. **What is the pathogenesis of chromoblastomycosis infection?**

## DISCUSSION

**Figure 1:**
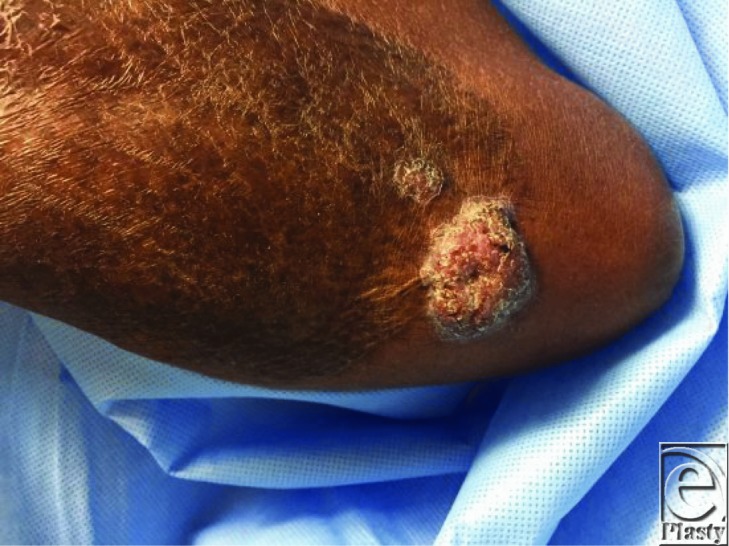
Cauliflower-like mass at the edge of split-thickness skin graft.

**Figure 2:**
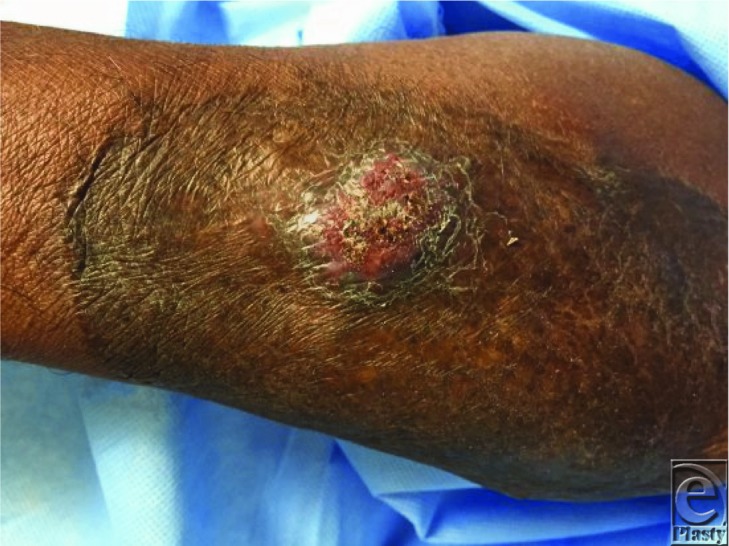
Second lesion that is more plaque-like in character with ulceration.

Chromoblastomycosis is a subcutaneous infection caused by dematiaceous fungi endemic to equatorial regions. This condition presents in early stages as verrucous nodules, which may progress to cauliflower-like tumors or large plaques with ulceration (Figs 1 and 2).^1^ In later stages of disease, dissemination of fungus through lymphatic migration or autoinoculation with scratching may result in progression of lesions, development of new lesions, and secondary infections.^2^ In severe cases, chronic lymphedema may mimic elephantiasis. Rarely, squamous cell carcinoma can develop in the setting of chronically inflamed tissue.^3^ The differential diagnosis for chromoblastomycosis includes infections such as tuberculosis, papillomavirus, leishmaniasis, and sporotrichosis, as well as psoriasis, chronic dermatitis, and malignancy. Ocular chromoblastomycosis may appear similar to melanoma.

Chromoblastomycosis is frequently refractory to multimodal therapy and has been historically difficult to manage. Treatment options include cryotherapy, topical heat therapy, surgical excision, and systemic medications. Itraconazole, 200 to 400 mg for 8 to 12 months, is the current antifungal agent of choice, although fluconazole, terbinafine, and amphotericin have been used with success.^4^ There is a paucity of literature regarding surgical excision of chromoblastomycosis. Several case studies report full remission first by treating with preoperative antifungal therapy to shrink the lesion, followed by surgical excision with negative margins.^5^

**Figure 3:**
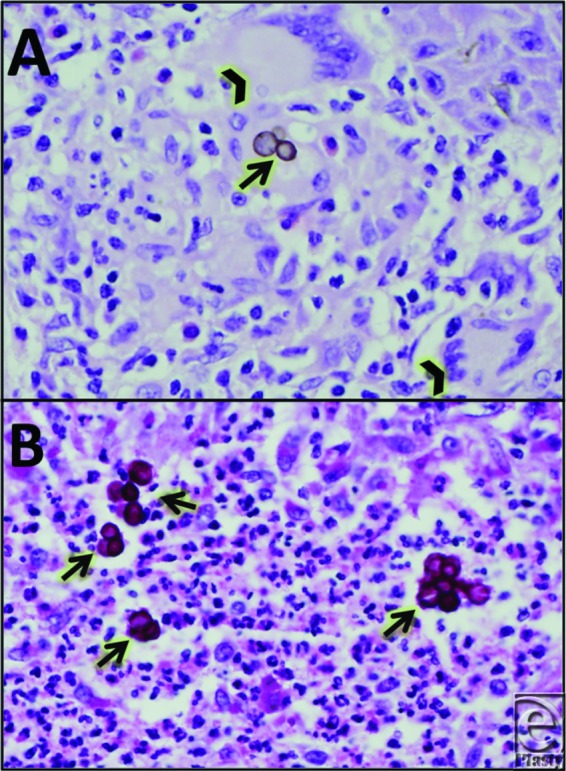
(a) Hematoxylin and eosin staining shows thick-walled, brown sclerotic bodies (arrow) in association with microabscess and multinucleated giant cells (arrowhead), consistent with chromoblastomycosis. (b) Periodic acid-Schiff-diastase stain highlights characteristic thick-walled sclerotic bodies (arrow).

Chromoblastomycosis is diagnosed on the basis of clinical examination and laboratory analysis. Microscopic examination of skin scrapings on potassium hydroxide (KOH) slide mounts reveal dark-pigmented, round, thick-walled, multiseptate, muriform cells approximately 10 to 14 μm in diameter, often in clusters, that are pathognomonic for chromoblastomycosis. These cells are eponymously referred to as “Medlar bodies” or “copper pennies” (Fig 3). Tissue culture in Sabouraud agarose grows out velvety colonies in approximately 2 weeks that can help with species identification. More recently, serum antigenic analysis with enzyme-linked-immunosorbent serologic assay technology, which is quite specific, has become more available and is utilized in endemic regions.^6^

The pathogenesis of chromoblastomycosis involves inoculation of soil-based fungi into the dermis and subcutaneous tissue via puncture injury and is most commonly found in men aged 30 to 50 years who work in farming and agriculture. It is less common in women; female hormone production is suggestive of a protective effect. The HLA-A29 haplotype is associated with an increased risk of infection, suggesting a genetic immune susceptibility to fungal infection for some patients. Hematoxylin-eosin slides of biopsy tissue reveal granular infiltrates, epithelial pseudohyperplasia with keratosis, fibrosis, acanthosis, and papillomatosis, suggestive of a chronic cutaneous inflammatory process.^7^ The drivers of host defense in chromoblastomycosis are poorly understood, although both cellular and humoral activities are thought to play important roles.^8^ The time from inoculation to clinical symptoms may be years and is determined by the accumulation of local tissue sequelae from ongoing inflammation.
